# Selecting the Appropriate Radiation Therapy Technique for Extensive Brain Metastases from Tens to Hundreds: Should the Latest Technique Always Be the Best Option?

**DOI:** 10.3390/medicina59101815

**Published:** 2023-10-12

**Authors:** Seok Ho Lee

**Affiliations:** Department of Radiation Oncology, Gil Medical Center, Gachon University College of Medicine, Incheon 21565, Republic of Korea; souko@gilhospital.com

**Keywords:** brain metastases, whole-brain radiotherapy (WBRT), stereotactic radiosurgery (SRS), intensity-modulated radiotherapy (IMRT)

## Abstract

Brain metastases (BMs) are one of the most common metastatic lesions in adult cancer patients and the most common intracranial neoplasms in adult patients. Especially for multiple BMs, historically, whole-brain radiotherapy (WBRT) has been performed as the mainstay of therapy, which improves neurological symptoms and median survival. However, WBRT could negatively impact the patient’s quality of life due to late complications. Owing to these complications, attempts have been made to use the latest radiotherapy (LRT) such as stereotactic radiosurgery (SRS) and intensity-modulated radiotherapy (IMRT) to treat BMs. However, for the extensive BMs (ranging from tens to hundreds), there are currently no prospective studies comparing WBRT with LRT such as IMRT or SRS. For extensive brain metastases, LRT cannot be the best option. Instead, upfront WBRT should be considered given its advantages and disadvantages, rather than LRT. We hope that faster and more reliable LRT for extensive BMs will be applicable for clinical practice without any clinical concerns in the near future.

## 1. Introduction

Brain metastases (BMs) are one of the most common metastatic lesions in adult cancer patients and the most common intracranial neoplasms in adult patients [1,2]. Its incidence is likely to increase over time with increased surveillance and systemic control [3]. The most common causes of brain metastasis (BM) are lung cancer and breast cancer (5–20%) [4]. The most common sites of involvement in the brain are the cerebral hemisphere (approximately 80%), cerebellum (15%), and brainstem (5%) [5].

During the course of cancer, BMs are generally considered incurable disseminated disease. Moreover, BM is a more noticeable form of metastasis due to its symptoms and the need for urgent treatment. It may cause neurological damage in addition to headaches, a common presenting symptom that can lead to a dramatic deterioration in the patient’s quality of life, such as seizures, mental changes, and increased intracranial pressure. In particular, an increased intracranial pressure could result in brain herniation, one of the most devastating complications of BMs. Furthermore, 5–10% of patients may present with acute neurologic symptoms due to hemorrhage into the tumor or cerebral infarction from compressive or embolic occlusion of a cerebral vessel [5].

Therefore, immediate diagnostic workup and appropriate treatment should be performed when these symptoms are present. Although glucocorticoids are firstly indicated in symptomatic patients and improve neurological symptoms in most patients with cerebral edema, it is important to diagnose the early phase of BM and to treat it early on because symptomatic symptoms may be accompanied by neurologic deficits [4,6]. Therapeutic approaches to BM include surgery, whole-brain radiotherapy (WBRT), the latest radiotherapy (LRT) such as stereotactic radiosurgery (SRS), intensity-modulated radiotherapy (IMRT), chemotherapy [7], and supportive care. Many patients are treated with a combination of these, and treatment decisions must consider clinical prognostic factors to maximize survival and preserve neurologic function while avoiding unnecessary treatments based on multidisciplinary collaboration. Focusing on RT itself, the choice among these RT techniques depends on factors involving the patient (performance status, expected survival, and age), the tumor (histology, size, number, location, and extracranial disease status), and available treatment options (access to neurosurgery or SRS) [8].

**Figure 1:**
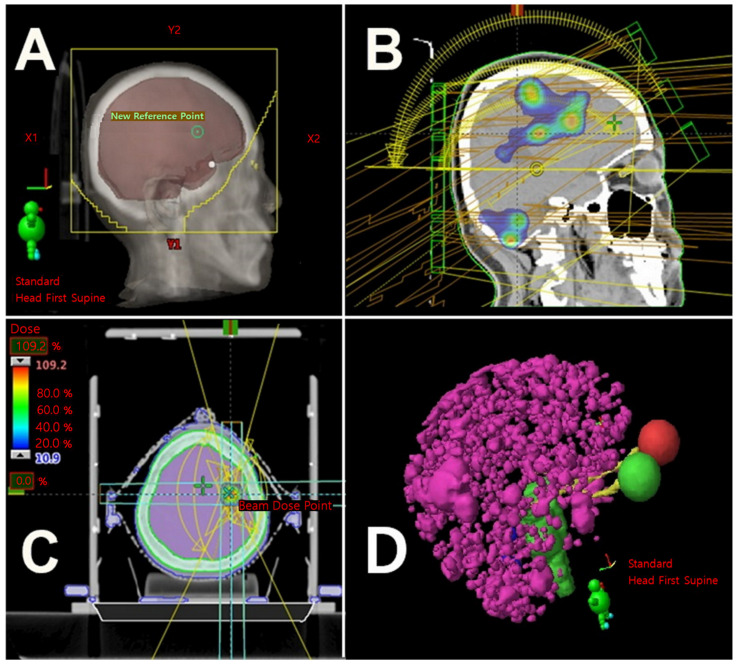
Whole-brain radiotherapy (**A**), volumetric modulated radiation therapy (**B**), stereotactic radiosurgery for brain metastases (**C**), and contouring for extensive brain metastases (ranging in the hundreds) (**D**).

Especially for multiple BMs, historically, WBRT has been performed as the mainstay of therapy, which improves neurological symptoms and median survival, from 1 to 2 months without WBRT, to 3 to 6 months with WBRT [9,10] (Figure 1). However, WBRT could negatively impact the patient’s quality of life due to late complications, such as cognitive dysfunction, which is reported in 50–90% of patients surviving more than 6 months after WBRT [11,12]. Owing to these complications, attempts have been made to use LRT, such as SRS and IMRT, including volumetric modulated arc radiotherapy (VMAT) with hippocampal sparing, to treat BM [13] (Figure 1). VMAT is an improved version of IMRT and can be considered a subset of IMRT. Among various LRTs, SRS can be performed on selective patients with oligometastases (up to four brain metastases), good performance status, controlled systemic disease, and even multiple lesions less than 3 to 4 cm [1,14,15,16,17]. It is well known that there are advantages of SRS over WBRT, such as limiting radiation to the normal brain and obtaining a higher local tumor control. Furthermore, in the study that enrolled patients with a life expectancy of 3–6 months and between one and ten BMs, the SRS arm showed a lower intracranial progression-free survival compared to the WBRT arm [18]. And, even in a limited number of asymptomatic BMs, repeated SRS to defer WBRT is performed in selected patients who relapse after SRS for BMs [19]. In patients with less than 10 metastatic lesions, large BMs with no neurologic symptoms, and well-controlled extracranial metastases, WBRT with simultaneous integrated boost using VMAT or SRS could be possible when considering the clinical benefits of reducing RT-related toxicities, such as preserving cognitive function and minimizing the interruption of systemic therapy, compared to WBRT [20,21]. Moreover, LRT such as SRS or IMRT could also be considered when salvage therapy is required during follow-up. Despite the many technical differences between LRT and WBRT, the reason for comparing the two techniques is the need to compare their efficacy in patients with brain metastases, especially those with extensive brain metastases. Therefore, in this study, we aimed to briefly state our opinions on the role of WBRT and LRT as radiotherapy modalities in patients with extensive BMs.

## 2. Rationale for and Concerns about the Use of LRT in Patients with Extensive Brain Metastases

Despite the LRT being implemented in patients with multiple BMs as described above, there is still no clear basis for applying it to patients, especially those with extensive BMs (ranging from tens to hundreds among the patients with multiple brain metastases). Nowadays, there are ongoing studies for comparing WBRT versus LRT for multiple BMs with less than a number of 10~15 metastatic lesions [22,23]. However, these studies enrolled the patients with brain metastases ranging less than tens or fifteens. There are no prospectively comparative studies between WBRT and LRT for the patients with brain metastases ranging from more than tens or fifteens to hundreds.

Nevertheless, due to the concers about the adverse impact of WBRT on neurocognition in these patients, some physicians are still tempted to use these state-of-the-art treatment techniques. Actually, LRTs are actually being used for extensive BMs in some institutions.

In general, compared to WBRT, LRT such as IMRT or SRS requires much longer time from the beginning of RT planning to the start of treatment. In the case receiving LRT in particular, the treatment plan processes for both contouring and RT planning and quality verification processes are essentially included. For example, if extensive BMs are present and their sizes are too small, it is important to consider whether LRT benefits are present in these patients. If IMRT or SRS is used in these patients, it takes considerable time and effort to define the extensive small targets through the fusion process of brain MRI and computed tomography for RT planning. In addition, if extensive tumor sizes are too small, it is questionable whether all of these tumors can be accurately defined.

## 3. Rationale for and Opinions on Performing WBRT in Patients with Extensive Brain Metastases

Therefore, in these patients, prompt treatment using WBRT can be preferable as the first RT option; especially in the situation whereby the neurological deficit has progressed but the RT is delayed and a limited survival is expected. Furthermore, WBRT as a prophylactic RT of the whole brain as a sanctuary was thought to be necessary because of the very high likelihood of new lesions developing in the brain [24].

As mentioned, a delay in treatment initiation may adversely affect the prognosis of the quality of life for some patients with BMs, especially those with neurological deficits. Even in the patients with extensive BMs, the chances of developing neurological death are expected to increase [25]. Furthermore, if appropriate patient selection is not achieved when using the LRT, the cost and hospital stay period for the patient may be increased without improving treatment efficiency. Therefore, the use of LRT should be carefully decided, considering the limitation of LRT, especially in symptomatic patients with extensive BMs.

Although the clinical benefits of LRT appear to be clear in patients with limited BMs, in multiple BMs, it has not yet been reported that LRT such as SRS is significantly superior to conventional WBRT for local control and patients’ quality life [26,27]. And, the benefits of the applicability of LRT to the patients with extensive BMs are still elusive, as described above. To emphasize once again, there are still no definite guidelines as to whether WBRT, IMRT, SRS, or WBRT with SRS should be performed first. From this point of view, the choice of LRT such as IMRT or SRS should be carefully determined especially in patients with extensive BMs. And, until now, upfront WBRT should be considered as a standard approach for multiple BMs; especially extensive BMs [28]. Along with that, WBRT can be a good and useful alternative for these patients who are being treated at radiotherapy centers in low- to middle-income countries with limited resources. For reference, the risk and number of brain metastases varies according to primary tumor type. For example, lung cancer and melanoma are frequently associated with multiple brain metastases, whereas in breast, colon, and renal-cell carcinoma, solitary brain metastases are more common. A representative study of the number of brain metastases reports the following. Studies using computed tomography and autopsy have reported a frequency of multiple brain metastases, ranging from 58% to 86%, with an average of 66%, while studies using magnetic resonance imaging have reported 16%, 13%, 10%, and 40% for two, three, four, and five or more metastases, respectively, and 50% for three or more [29,30].

In this article, as a radiation oncologist, we wanted to mainly focus on the radiotherapeutic approach to the patients especially with extensive BMs, specifically according to the number of brain metastases, without dealing with topics such as chemotherapy, immunotherapy, and target agents, the issues of anatomical location, or the approach to surgical treatment.

## 4. Conclusions

In summary, there are currently no prospective studies comparing WBRT with LRT such as IMRT or SRS for extensive BMs (ranging from tens to hundreds). Therefore, there still remains an absence of guidelines for the personalized application of RT techniques for these patients. Despite the evidence of the efficacy of LRT on patients with limited BMs, its use is still controversial in patients with multiple BMs; especially extensive BMs. Accordingly, considering the rapid development of new lesions, the increase in the possibility of neurological death, and no evidence of LRT superiority over WBRT for these patients, the application of LRT to these patients should be examined and judiciously determined. Until now, it is believed that WBRT is preferable, considering its advantages and disadvantages rather than LRT, especially in the situation whereby the neurological deficit has progressed, but the prompt RT is delayed and a limited survival is expected. Owing to the rapid development of radiotherapy software and hardware as cutting-edge RT techniques, we hope that faster and more reliable LRTs for extensive BMs will be applicable for clinical practice without any clinical concerns in the near future.

## Data Availability

Not applicable.
